# A new nomogram to predict the need for tracheostomy in burned patients

**DOI:** 10.1007/s00405-020-06541-3

**Published:** 2020-12-21

**Authors:** Stefan Janik, Stefan Grasl, Erdem Yildiz, Gerold Besser, Jonathan Kliman, Philipp Hacker, Florian Frommlet, Alexandra Fochtmann-Frana, Boban M. Erovic

**Affiliations:** 1 Department of Otorhinolaryngology, Head and Neck Surgery, Medical University Vienna, Vienna, Austria; 2 Center for Medical Statistics, Informatics, and Intelligent Systems, Medical University of Vienna, Vienna, Austria; 3 Division of Plastic and Reconstructive Surgery, Department of Surgery, Medical University Vienna, Vienna, Austria; 4 Institute of Head and Neck Diseases, Evangelical Hospital Vienna, Hans-Sachs Gasse 10-12, Vienna, Austria

**Keywords:** Dysphagia, Tracheostomy, Complications, Nomogram, Burn injury

## Abstract

**Purpose:**

To evaluate the impact of tracheostomy on complications, dysphagia and outcome in second and third degree burned patients.

**Methods:**

Inpatient mortality, dysphagia, severity of burn injury (ABSI, TBSA) and complications in tracheotomized burn patients were compared to (I) non-tracheotomized burn patients and (II) matched tracheotomized non-burn patients.

**Results:**

134 (30.9%) out of 433 patients who underwent tracheostomy, had a significantly higher percentage of inhalation injury (26.1% vs. 7.0%; *p* < 0.001), higher ABSI (8.9 ± 2.1 vs. 6.0 ± 2.7; *p* < 0.001) and TBSA score (41.4 ± 19.7% vs. 18.6 ± 18.8%; *p* < 0.001) compared to 299 non-tracheotomized burn patients. However, complications occurred equally in tracheotomized burn patients and matched controls and tracheostomy was neither linked to dysphagia nor to inpatient mortality at multivariate analysis. In particular, dysphagia occurred in 6.2% of cases and was significantly linked to length of ICU stay (OR 6.2; *p* = 0.021), preexisting neurocognitive impairments (OR 5.2; *p* = 0.001) and patients’ age (OR 3.4; *p* = 0.046). A nomogram was calculated based on age, TBSA and inhalation injury predicting the need for a tracheostomy in severely burned patients.

**Conclusion:**

Using the new nomogram we were able to predict with significantly higher accuracy the need for tracheostomy in severely burned patients. Moreover, tracheostomy is safe and is not associated with higher incidenc of complications, dysphagia or worse outcome.

## Introduction

Airway management and subsequently patients’ safety are crucial in treatment of severely burned patients. While endotracheal intubation is performed to secure airway, tracheostomy is selectively performed when prolonged ventilation support is expected due to the extent of burn injury itself or other comorbidities [1, 2].

Tracheostomy may be performed either as surgical tracheostomy (ST) or as percutaneous dilatational tracheostomy (PDT). In general, tracheostomy provides several advantages such as reduction of dead space and airway resistance, ventilation weaning, reduction of required sedation and thereby enabling earlier active rehabilitation and safe airway management during daily wound and tracheobronchial care [3, 4].

Apart from these major advantages, tracheostomy was also considered to be linked to development of dysphagia [5]. In non-tracheotomized burn patients, length of orotracheal intubation significantly affects occurrence of dysphagia [6] and therefore, indication and timing for tracheostomy have been discussed extensively in recent literature [7–9]. Although favorable effects of early tracheostomy in severely burned patients were reported, studies still failed to demonstrate beneficial effects of early tracheostomy on inpatient mortality, length of inpatient stay or overall survival [7, 8].

Hypothesizing that tracheostomy may have a negative impact on the incidence of dysphagia in severely burned patients, we set up this study to determine the association between performance of tracheostomy on the occurrence of dysphagia and inpatient mortality. Moreover, we assessed and compared the incidence of tracheostomy-related complications in burn patients to matched tracheotomized non-burn patients in order to further prove the safety of this procedure in a large, homogenous cohort. Based on our data, we developed a nomogram to predict the likelihood for tracheostomy in severely burned patients.

## Materials and methods

### Study cohort

We conducted a retrospective cohort study including 433 burn patients with presence of deep partial- to full-thickness (IIb–III) thermal injuries who were admitted to the burn ICU of the General Hospital Vienna, Austria, for at least 24 h. All patients were treated between 01/2008 and 12/2016. We used the abbreviated burn severity index (ABSI) and the total body surface area (TBSA) score to graduate severity of burn injury. Patients with suspected inhalation injury underwent flexible transnasal laryngoscopy by an ENT physician. Presence of carbonaceous sputum and signs of airway obstruction, such as edema, were used as an indicator for inhalation injury [10].

### Clinical data

Sociodemographic and clinical data were retrospectively collected from electronic patient records (Table 1). Emphasizing on functional swallowing outcome and dysphagia as one of the main outcome parameters, we systematically screened patients’ histories for the presence of preexisting neurocognitive impairments (e.g., dementia, insult). Patients were followed until discharge from burn ICU or death.

**Table 1:** Study cohort

Clinical characteristics	Burn patients			
	Total	Tracheostomy		*p*
		Yes	No	
Sex	433 (100)	134(30.9)	299 (69.1)	
Male	271 (62.6)	83 (30.6)	188 (69.4)	
Female	162 (37.4)	51 (31.5)	111 (68.5)	0.915
Age				
Mean (median) ± SD	49.2 (48.0) ± 21.2	49.7 (49.5) ± 20.3	49.0 (46.0) ± 21.7	0.730
Inhalation injury				
Yes	56 (12.9)	35 (62.5)	21 (37.5)	
No	377 (87.1)	99 (26.3)	278 (73.7)	< 0.001
Burn of head and neck				
Yes	208 (48.0)	86 (41.3)	122 (58.7)	
No	172 (39.7)	47 (27.3)	125 (72.7)	
Unknown	53 (12.2)	1 (1.9)	52 (98.1)	< 0.001
ABSI				
Mean (median) ± SD	6.9 (7.0) ± 2.9	8.9 (9.0) ± 2.1	6.0 (5.0) ± 2.7	< 0.001
TBSA				
Mean (median) ± SD	25.6 (20.0) ± 21.8	41.4 (40.0) ± 19.7	18.6 (12.0) ± 18.8	< 0.001
Reason for burn injury				
Combustion	202 (46.7)	86 (42.6)	116 (57.4)	
Scald	83 (19.2)	12 (14.5)	71 (85.5)	
Explosion	52 (12.0)	18 (34.6)	34 (65.4)	
Electric burn	22 (5.1)	9 (40.9)	13 (59.1)	
Unknown	74 (17.1)	–	–	< 0.001
Length of ICU stay (days)				
Mean (median) ± SD	23.1 (12.0) ± 29.5	49.1 (38.0) ± 37.9	11.6 (6.0) ± 13.8	< 0.001
Nutrition via NGT (days)				
Mean (median) ± SD	29.2 (19.5) ± 32.4	36.7 (29.0) ± 32.0	11.2 (6.0) ± 25.5	< 0.001
Dysphagia				
Yes	23 (5.3)	14 (60.9)	9 (39.1)	
No	348 (80.4)	120 (34.5)	228 (65.5)	
Unknown	62 (14.3)	–	–	0.014
Inpatient mortality				
No	353 (81.5)	97 (27.5)	256 (72.5)	
Yes	80 (18.5)	37 (46.2)	43 (53.8)	
MODS	46 (10.6)	23 (50.0)	23 (50.0)	
Cardiopulmonary Dysfunction	17 (3.9)	3 (17.6)	14 (82.4)	
Sepsis	9 (2.1)	6 (66.7)	3 (33.3)	
Unknown/other	8 (1.8)	5 (62.5)	3 (37.5)	< 0.001

*ABSI* abbreviated burn severity index, *TBSA* total body surface area, *NGT* nasogastric tube, *MODS* multiorgan dysfunction syndrome, *SD* standard deviation

### Performance and complications of tracheostomy

Number of tracheostomies and whether they were surgically or percutaneously done was evaluated. Subsequently the onset of complications was determined. Since percutaneous tracheostomies (PDT) were rarely performed (*n* = 12) at our institution, we solely analyzed complications in patients who underwent surgical tracheostomy with creation of an inferiorly based, U-shaped flap [11].

Complications were stratified into the following groups: (I) bleeding that needs management in the OR; (II) persistent tracheostomy: defined as persistence of epithelized stoma longer than 4 weeks after decannulation; (III) wound infection/dehiscence with need of surgical revision; (IV) tracheal stenosis.

To better estimate the impact of burn injury on the occurrence of complications, we compared tracheostomy-related complications in burn patients to a cohort of sex-and age-matched tracheotomized non-burn controls who underwent surgical tracheostomy due to other clinical issues. Data of this control cohort were extracted from one of our previous works [12].

### Dysphagia

Fiberendoscopic evaluation of swallowing (FEES) or modified barium swallowing (MBS) test are commonly used for evaluation of dysphagia [13]. In accordance to literature [14], we used the penetration-aspiration-scale (PAS) score differentiating between normal swallowing (PAS 1), penetration (PAS 2–5), and aspiration (PAS 6–8) to classify swallowing results [15]. Patients with PAS scores > 3 received speech and language therapy for swallowing rehabilitation. Those patients with clinical signs of aspiration (PAS 6–8) remained nil per os (NPO) and were nourished only through nasogastric tube (NGT) feeding. Other reasons for NGT feeding were prolonged sedation with absent consciousness and insufficient oral intake. We classified patients as being dysphagic if they did not adequately improve during swallowing recovery, either due to aspiration or persistent insufficient oral intake.

### Inpatient mortality

Inpatient mortality was defined as death occurring during inpatient stay. Although reasons for inpatient mortality are often overlapping, we allocated reasons for death according to the following causes: (I) multiple organ dysfunction syndrome (MODS) [16]; (II) sepsis [17]; (III) cardiorespiratory dysfunction. Patients were stratified to the cardiorespiratory dysfunction group if they predominantly died from cardiorespiratory dysfunction instead of MODS or sepsis.

### Statistical methods

Statistical analyses were performed using SPSS version 26.0 software (IBM SPSS Inc., Armonk, NY, USA) and R version 3.6.0 (R Core Team, 2019; R Foundation for Statistical Computing, Vienna, Austria). Unless otherwise specified, data are reported as mean ± standard deviation (SD). Descriptive statistics were used for analysis of demographic and clinical data. Chi-Square test and independent-students *T*-test were applied to compare nominal variables and to analyze means of two normally distributed variables, respectively. Univariate and multivariate binary regression analyses were used to evaluate the impact of age (≥ 48y), sex (female), performance of tracheostomy (Trach vs. non-Trach), presence of dysphagia (yes vs. no), duration of ICU stay (≥ 12 days), nutrition through NGT (NGT ≥ 19.5 days), preexisting neurocognitive impairment (yes vs. no), burn injury of head and neck (yes vs. no), presence of inhalation injury (yes vs. no), ABSI (high vs. low) and TBSA score (high vs. low) on inpatient mortality and dysphagia, respectively. The median was used for metric variables (e.g. age, TBSA, etc.) for dichotomizing patients into subgroups. Odds Ratios (ORs) and corresponding 95% confidence intervals (CIs) are indicated. All tests were performed two-sided and *p* values below 0.05 were considered as statistically significant.

In order to create a nomogram, we performed variable selection among all potential predictor variables and some pairwise interactions using logistic regression models. Stepwise backward elimination based on the Akaike information criterion (AIC) was applied to obtain the final best multivariable logistic regression model, which was visualized with a nomogram using the R package “rms” [18].

### Ethical consideration

Ethical approval was obtained from the ethical review board of the Medical University of Vienna (EK No. 1758/2017).

## Results

### Study cohort

A total of 433 burn patients, including 271 males (62.6%) and 162 females (37.4%), with a median age of 48.0 ± 21.2 years were analyzed. Burn injuries were caused by combustion, scald, explosion and electric burn in 56.3%, 23.1%, 14.5%, and 6.1% of cases, respectively. Inhalation injury was diagnosed in 56 patients (12.9%) and the median ABSI and TBSA score were 7.0 ± 2.9 and 20.0 ± 21.9%, respectively. Burn patients were treated at ICU for 23.1 ± 29.5 days ranging from 1 to 182 days (Table 1).

### Tracheostomy in burn patients

**Fig. 1:**
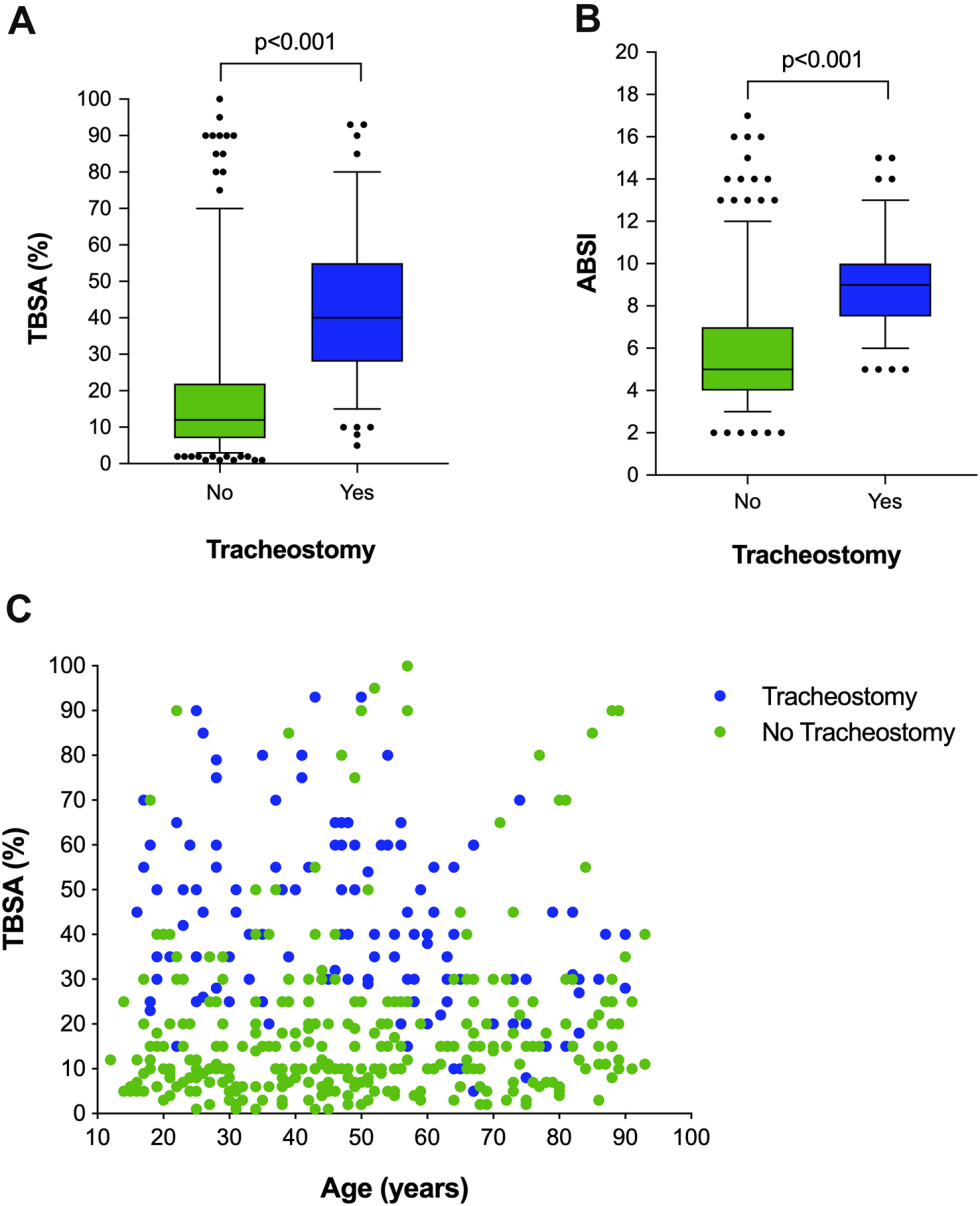
Tracheostomy in burn patients. Tracheostomy was performed in patients with significantly higher TBSA (**a**) and ABSI (**b**) scores, respectively. Moreover, there was strong interaction between extent of burn injury, indicated by TBSA score, age and performance of tracheostomy (**c**)

Out of 134 patients who underwent tracheostomy (30.9%), 122 (91.0%) and 12 (9.0%) patients underwent ST and PDT, respectively. Mean time between admission and performance of tracheostomy was 1.4 ± 3.8 days and did not significantly differ between tracheostomy techniques (ST vs. PDT 1.4 ± 4.0 vs. 2.6 ± 2.4 days; *p* = 0.319). ABSI (8.9 ± 2.1) and TBSA score (41.4 ± 19.7%) in tracheotomized burn patients were significantly higher compared to non-tracheotomized patients (ABSI 6.0 ± 2.7; *p* < 0.001; TBSA 18.6 ± 18.8%; *p* < 0.001; Fig. 1a, b).

Tracheotomized burn patients experienced significantly more often inhalation injuries (26.1% vs. 7.0%; *p* < 0.001), were younger and had higher TBSA scores (Fig. 1c). The same patients showed a prolonged ICU stay (49.1 ± 37.9 days) and NGT feeding (36.7 ± 32.0 days) compared to non-tracheotomized burn patients (11.6 ± 13.8 days; *p* < 0.001 and 11.2 ± 25.5 days; *p* < 0.001; Table 1). Similarly, inpatient mortality was 1.5 times higher in burn patients with tracheostomy compared to those without (24.4% vs. 16.3%; *p* = 0.073).

### Incidence and risk factors for the development of dysphagia

Data regarding nutrition was available in 371 (85.7%) out of 433 patients and dysphagia was diagnosed in 23 (6.2%) patients. Patients were fed with the NGT in 176 (47.4%) cases with a median time of 19.5 ± 32.4 days. However, dysphagia occurred significantly more often in patients (i) suffering from preexisting neurocognitive impairment (*p* < 0.001), (ii) with tracheostomy (*p* = 0.014), (iii) with high ABSI (*p* < 0.001), and (iv) older than 48 years (*p* < 0.001). Duration of intubation or tracheostomy had no significant impact on the development of dysphagia (*p* = 0.456). At multivariate logistic regression analyses (Table 2), cases with ICU stays longer than 12 days, preexisting neurocognitive impairment and age ≥ 48 years were associated with a 6, 5, and 3 times higher risk for the development of dysphagia (OR 6.2; *p* = 0.021; OR 5.2; *p* = 0.001; OR 3.4; *p* = 0.046), respectively.

**Table 2:** Dysphagia

Variables	Univariate analysis			Multivariate analysis		
	OR	*p*	95% CI	OR	*p*	95% CI
Age (≥ 48 years)	5.6	0.002	1.9–16.9	3.4	0.046	1.0–11.2
Sex (female)	1.3	0.558	0.6–3.0			
Trach vs. burn	3.0	0.014	1.2–7.0	1.3	0.650	0.4–4.5
NGT (≥ 19.5 days)	2.2	0.110	0.8–5.8			
Neurocognitive impairment (yes)	8.1	< 0.001	3.2–20.4	5.2	0.001	2.0–13.7
ICU stay (≥ 12 days)	7.0	0.002	2.1–23.8	6.2	0.021	1.3–29.4
Burn of head and neck	0.8	0.789	0.4–2.1			
Inhalation injury	0.7	0.581	0.2–2.9			
TBSA (high)	1.5	0.318	0.7–3.6			
ABSI (high)	3.9	0.003	1.6–9.4	1.4	0.623	0.4–4.5

Uni- and multivariate logistic regression analyses were performed to evaluate the impact of different clinical variables on the development of dysphagia. The median age (48 years), duration of nutrition through the nasogastric feeding tube (NGT, 19.5 days), ICU stay (12 days), total body surface area (TBSA) burn injury (20.0%), and ABSI (abbreviated burn severity index; 7.0) were used for dichotomizing patients into low and high subgroups

### Surgical tracheostomy in burn patients compared to controls

To analyze the incidence of complications and outcome in surgically tracheotomized burn patients, we compared data of burn patients who were tracheotomized to an age- (54.1 ± 19.8 vs. 54.3 ± 19.6 years) and sex- (F:M ratio; 45:65 vs. 44:66) matched control cohort of tracheotomized, non-burn patients (*p* = 0.932; *p* = 1.000).

Length of surgery was 36.7 ± 12.1 min in burn patients undergoing tracheostomy compared to 38.1 ± 14.2 min in patients of the control group (*p* = 0.447). Tracheostomies with elevation and preservation of thyroid isthmus, determined as ‘low-tracheostomies’, were performed in 19 (25.3%) burn patients and 32 (31.1%) controls (*p* = 0.502; Table 3).

**Table 3:** Complications in tracheotomized burn patients compared to matched controls

Variables	Total *n* (%)	Surgical tracheostomy		*p*
		Burn *n* (%)	Control *n* (%)	
Sex	220 (100.0)	110 (50.0)	110 (50.0)	
Male	131 (59.5)	65 (49.6)	66 (50.4)	
Female	89 (40.6)	45 (50.6)	44 (49.4)	1.000^a^
Age				
Mean (median) ± SD	54.2 (56.7) ± 19.7	54.1 (56.6) ± 19.8	54.3 (56.8) ± 19.6	0.932^b^
Length of surgery				
Mean (median) ± SD	37.4 (35.0) ± 13.2	36.7 (35.0) ± 12.1	38.1 (35.0) ± 14.2	0.447^b^
Complications				
No	202 (91.8)	101 (50.0)	101 (50.0)	
Yes	18 (8.2)	9 (50.0)	9 (50.0)	1.000^a^
Persistent stoma	11 (5.0)	7 (63.6)	4 (36.4)	
Bleeding	5 (2.3)	1 (20.0)	4 (80.0)	
Wound revision	1 (0.5)	1 (100.0)	0 (0.0)	
Tracheal stenosis	1 (0.5)	0 (0.0)	1 (100.0)	0.202^a^

*SD* standard deviation

^a^Chi-square test

^b^Independent students *T*-test

Complications occurred equally (*n* = 9) in both groups (8.2% vs. 8.2%; *p* = 1.000). A persistent tracheostoma was the predominant complication found in burn patients (*n* = 7) followed by bleeding (*n* = 1) and stomal infection with necessity for surgical revision (*n* = 1). Although differences failed to reach statistical significance (*p* = 0.202), persistent tracheostoma occurred 1.7 times more often in burn patients compared to non-burn patients (Table 3).

### Inpatient mortality

**Fig. 2:**
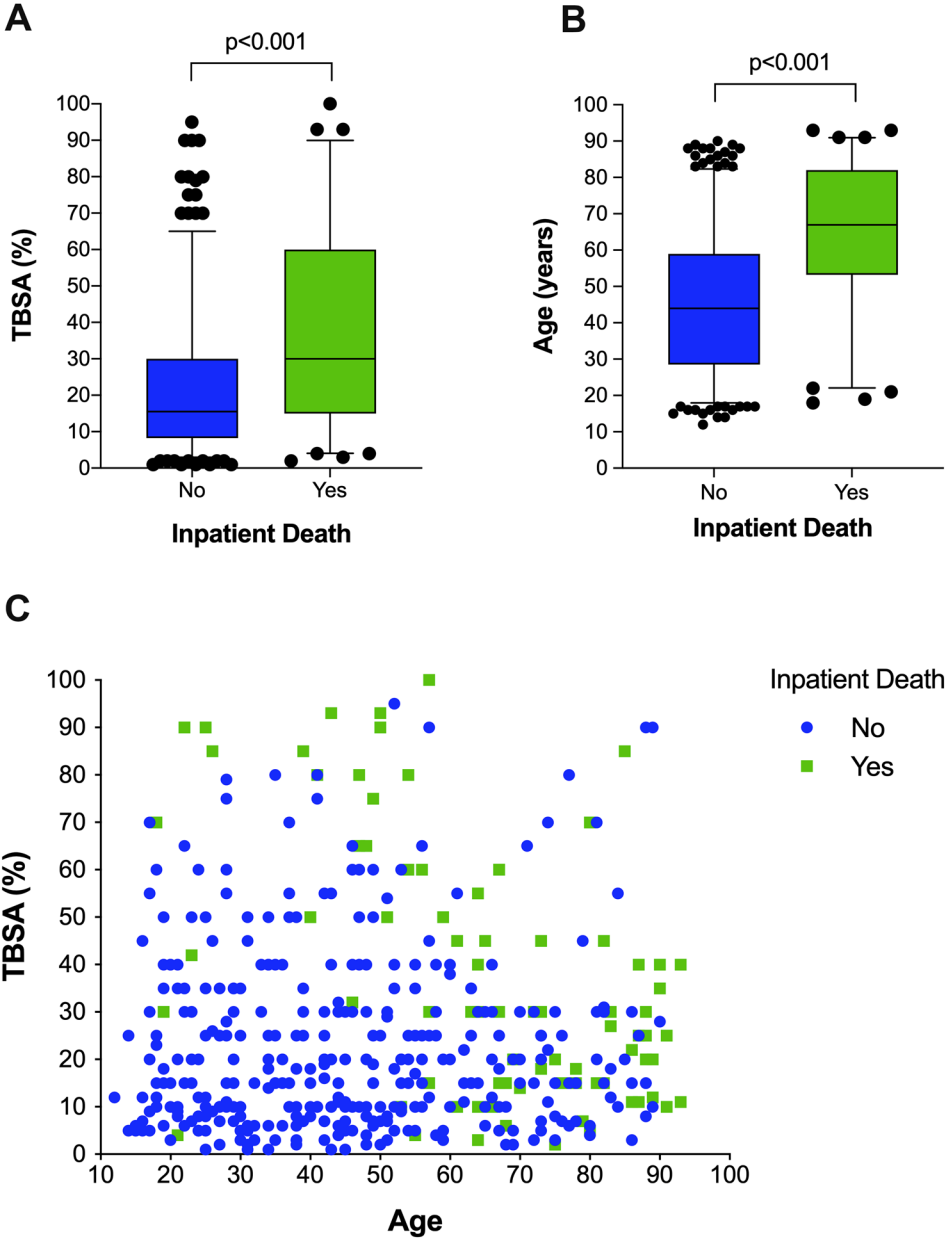
Inpatient death. Patients who died during inpatient stay had significantly higher TBSA scores (**a**) and were significantly older (**b**) compared to those who could be discharged after burn injury. The interaction between TBSA and age on inpatient death is charted (**c**). Particularly, if younger patients died during the inpatient stay, they had experienced higher burn injuries, while older patients died also with lower TBSA scores

A total of 80 (18.5%) burn patients died during inpatient stay. The majority of these patients died from MODS followed by cardiopulmonary dysfunction and sepsis in 10.6%, 3.9%, and 2.1% of cases, respectively (Table 1). Patients who died during inpatient stay had significantly higher TBSA scores (38.1 ± 27.6% vs. 22.8 ± 19.1%; *p* < 0.001) and were significantly older (65.2 ± 19.9 vs. 45.6 ± 19.8 years; *p* < 0.001). The interaction between TBSA score, age and inpatient death is shown in Fig. 2. As shown in Table 4, high ABSI (OR 7.1; *p* < 0.001), patients´ age ≥ 48 years (OR 4.0; *p* < 0.001) and presence of inhalation injury (OR 2.5; *p* = 0.029) represented independent prognosticators for death during inpatient stay.

**Table 4:** Inpatient mortality in regard to different clinical variables

Variables	Univariate analysis			Multivariate analysis		
	OR	*p*	95% CI	OR	*p*	95% CI
Age (≥ 48 years)	6.3	< 0.001	3.4–11.6	4.0	< 0.001	1.8–8.6
Sex (female)	2.0	0.005	1.2–3.3	1.1	0.815	0.6–2.1
Trach vs. burn	2.3	0.001	1.4–3.7	0.7	0.367	0.3–1.7
Dysphagia (yes)	3.8	0.002	1.6–9.2	1.7	0.312	0.6–4.8
NGT (≥ 19.5 days)	0.9	0.727	0.4–1.8			
Neurocognitive impairment (yes)	2.5	0.001	1.4–4.5	1.5	0.235	0.8–3.0
ICU stay (≥ 12 days)	1.1	0.653	0.7–1.8			
Burn of head and neck	1.1	0.806	0.6–1.8			
Inhalation injury	4.3	< 0.001	2.4–7.8	2.5	0.029	1.1–5.6
TBSA (high)	2.6	< 0.001	1.5–4.2	0.9	0.778	0.3–2.2
ABSI (high)	8.8	< 0.001	5.0–15.6	7.1	< 0.001	2.5–20.0

Uni- and multivariate logistic regression analyses were performed to evaluate the impact of different clinical variables on the development of dysphagia. The median age (48 years), duration of nutrition through the nasogastric feeding tube (NGT, 19.5 days), ICU stay (12 days), total body surface area (TBSA) burn injury (20.0%), and ABSI (abbreviated burn severity index; 7.0) were used for dichotomizing patients into low and high subgroups

### Nomogram

**Fig. 3:**
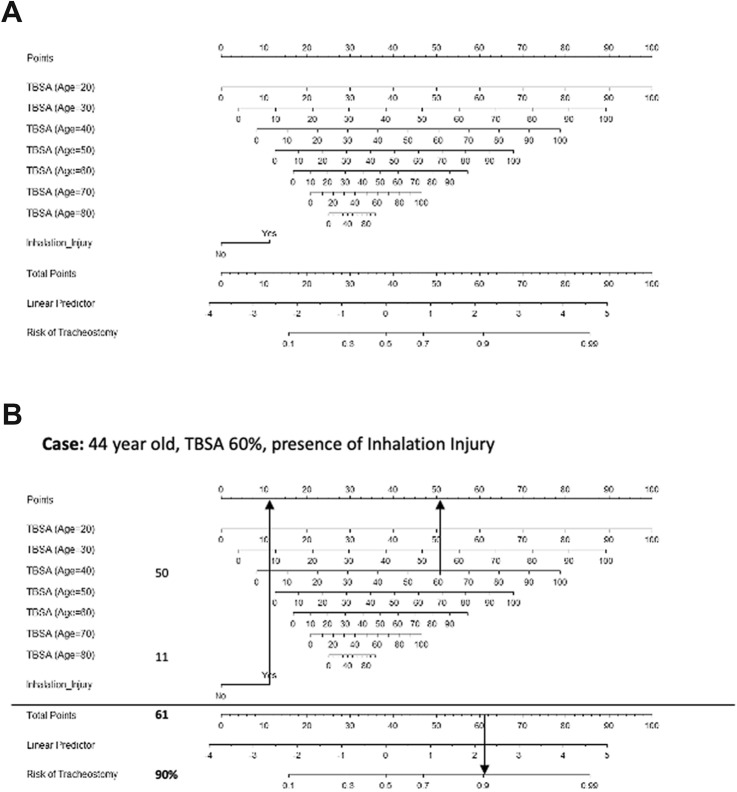
Nomogram to predict the need for tracheostomy. Age, TBSA score and inhalation injury were significant factors in our model (**a**). Due to the strong interaction effect of age and TBSA, the nomogram provides the scores for TBSA values stratified by age. Depending on the patients’ age, different TBSA scales need to be used. The TBSA (age = 20) scale can be used for patients aged 15–24 years, while TBSA (age = 30) can be used for patients aged 25 and 34 years, etc. As exemplified, a 44-year old patient with a TBSA of 60% and presence of inhalation injury has a 90% risk of tracheostomy (**b**)

After generating all clinical data retrospectively, we calculated a nomogram (AIC = 431.0; *R*^2^  = 0.25) to predict the probability for performance of tracheostomy in severely burned patients (Fig. 3). Thereby, age, TBSA and presence of inhalation injury were identified as significant factors. The strong interaction between age and TBSA is also represented in our model, where different TBSA scores are provided for specific age ranges. For patients aged 15–24 years, the TBSA (age = 20) scale has to be applied, while for patients aged 25–34 years, the TBSA (age = 30) scale has to be applied etc. Altogether, our nomogram illustrates that severity of burn injury, indicated by the TBSA score and age are the key factors for clinicians to decide whether or not the patient specifically needs tracheostomy.

## Discussion

In the most recent publications burn patients received tracheostomy in 4.3–23% of cases [6–8, 19–25]. Our study provides data of clinical outcome and complications of 134 (30.9%) tracheotomized burn patients and therefore represents, to our knowledge, one of the largest single center experiences.

According to our data, tracheostomy was particularly performed in patients with advanced burn injury. As a consequence of the severity of burn injury and its significant systemic contribution [7], ICU stay was 4.2 times longer compared to non-tracheotomized patients. Consequently, inpatient mortality was also 1.5 times higher in tracheotomized compared to non-tracheotomized burn patients and more frequently caused by MODS and sepsis. It was obvious that inpatient mortality was linked to advanced patients’ age, inhalation injury and high ABSI but, most importantly, tracheostomy was not associated with clinical outcome.

Moreover, safety of tracheostomy in pediatric and adult burn patients has been well reported in literature [2, 3, 22]. However, to better analyze and quantify the effect of burn injury on the occurrence of short-term complications, we compared the outcome of tracheotomized burn patients to tracheotomized matched controls who had been analyzed in one of our previous works [12]. Importantly, complication rates were equal in both subgroups but lower in our cohort compared to current literature reporting complications in 9% to 16% of burn patients [3, 22, 25]. Subsequently, tracheostomy performed in burn patients does not automatically lead to a higher incidence or more severe short-term complications. Although a persistent stoma was reported as the most common complication, we did not observe significantly higher numbers of chest infections as previously speculated to be caused by tracheostomy in burn patients [2].

Due to the short observation period that typically ends with discharge from the ICU, we could not provide data on long-term complications. Previous works reported high incidences of major complications, such as tracheoesophageal or tracheo-innominate artery fistulas or tracheal stenoses [8, 21, 26]. However, more recent studies refute these findings, reporting minor complications and only occasionally occurring cases of tracheal stenoses [3, 8, 22, 25]. Consistent with recent literature, there seems to be no significant difference in short- and long-term complications between burn patients and other critically ill patients who require tracheostomy.

Beside tracheostomy and airway management, many studies evaluated functional outcome parameters in burn patients. Interestingly, Clayton et al. found an increased risk of dysphagia in tracheotomized burn patients. Whether dysphagia was directly caused by tracheostomy, prolonged transoral intubation or inhalation injury is still unclear [19]. The incidence of dysphagia was 16 times higher in burn patients with inhalation injury compared to those without [18]. The proposed underlying mechanisms for developing dysphagia are oropharyngeal muscle disuse resulting in atrophy [27, 28] and impaired oral, pharyngeal and laryngeal mucosa sensory due to inhalation injury [18, 19, 29]. This is further supported by Smailes et al. demonstrating that the most predictive factor of dysphagia was prolonged duration of translaryngeal intubation and ventilation prior to tracheostomy. Particularly, the probability for developing dysphagia increased significantly if tracheostomy was performed more than 7 days after transoral intubation [3]. Despite former studies failed to show beneficial effects of early tracheostomy in regards to inpatient mortality and ventilation support [7, 8], some authors nevertheless recommend early tracheostomy to reduce the risk of dysphagia [3].

We observed dysphagia in 6.3% of our cases, which was lower compared to published rates ranging from 11.2% to 89.5% in similar cohorts [19, 29]. Advanced patient age, prolonged ICU stay and known neurocognitive impairment were negative prognosticators for the occurrence of dysphagia in our study. The importance of neurocognitive health for swallowing recovery was already accentuated in literature [19, 30]. We hypothesize that different definitions of dysphagia, the inconsistent use of assessment tools and the heterogeneity of tested patient cohorts regarding burn injury and tracheostomy may be responsible for divergent reports regarding incidence of dysphagia in burn patients. Moreover, tracheostomies were performed in the majority of our patients within the first 48 h of admission to burn ICU, which is significantly faster compared to cases in recent literature, with tracheostomies mostly performed within the 7th and 14th day of admission [1, 2, 31]. Again these data underline that early tracheostomy may help to reduce the risk of dysphagia in severely burned patients [3].

Based on our data, we further created a nomogram to predict the need for tracheostomy in second and third degree burn patients. As outlined above, there was a strong link between TBSA and age and the indication for tracheostomy. Although a higher amount of burn injury was principally linked to tracheostomy, tracheostomy was less commonly performed in older patients. We assume that physicians tried to avoid an escalation of treatment and subsequently avoided tracheostomy in advanced-aged patients with poor estimated prognosis.

We believe that the strength of this study lies in the large sample size as well as in the development of the first nomogram for tracheostomy in severely burned patients. However, we see three limiting factors. First, our study was conducted as retrospective analysis and bears therefore an inherent risk of information bias. Second, we could not provide long-term complication rates of burn patients due to the short follow-up period. Third, burn patients are not routinely evaluated for signs of dysphagia and subsequently the number of dysphagic patients might have been even higher.

## Conclusion

Patients requiring tracheostomy showed more extensive burn and inhalation injuries. But, however, tracheostomy is a safe procedure and does not cause more complications or dysphagia. Moreover, dysphagia itself was significantly associated with preexisting neurocognitive impairment, advanced patients’ age and prolonged ICU stay. Future studies will show the accuracy of the new nomogram in clinical practice for predicting the need for tracheostomy in burned patients.
